# Developing a Web-Based Version of An Exercise-Based Rehabilitation Program for People With Chronic Knee and Hip Pain: A Mixed Methods Study

**DOI:** 10.2196/resprot.5446

**Published:** 2016-05-19

**Authors:** Jennifer Pearson, Nicola Walsh, Desmond Carter, Sian Koskela, Michael Hurley

**Affiliations:** ^1^ School of Rehabilitation Sciences Faculty of Health, Social Care and Education St Georges University of London and Kingston University London United Kingdom; ^2^ Centre for Health and Clinical Research Department of Allied Health Professions University of the West of England Bristol United Kingdom; ^3^ Health Innovation Network NHS London United Kingdom; ^4^ Health Innovation Network South London NHS England London United Kingdom

**Keywords:** osteoarthritis, exercise, self-care, web-based program, behavioral change, qualitative research, quantitative research

## Abstract

**Background:**

Osteoarthritis is highly prevalent and has enormous personal and socioeconomic impact. Enabling Self-management and Coping with Arthritic Pain through Exercise (ESCAPE-pain) is an integrated rehabilitation program that helps people understand how exercise can improve physical and psychosocial well-being. Unfortunately, its availability is limited. A Web-based version of the program could increase access for more people. Many Web-based resources are developed without end-user input and result in over-complex, unwanted, ineffective products with limited uptake.

**Objective:**

The objective of this study was to codesign a Web-based version of ESCAPE-pain that people with chronic joint pain find engaging, informative, and useful.

**Methods:**

To establish older persons' Internet use we conducted a postal survey of 200 people. To establish their opinions, likes or dislikes, and requirements for a Web-based version of the ESCAPE-pain program, we conducted two focus groups with 11 people who had participated in a program based on ESCAPE-pain and two with 13 people who had not. Information from the postal survey and focus groups was used to develop an online prototype website. People's opinions of the prototype website were gauged from thematic analysis of eight semistructured “think aloud” interviews.

**Results:**

The survey response rate was 42% (83/200), of whom 67% (56/83) were female and mean age was 67 years. Eighty-three percent of the people had used the Internet, 69% described themselves as either very confident or confident Internet users, and 77% had looked online for health information. With regard to participating online, 34% had read a commentary or watched a video of someone else’s experience of a health problem and 23% had tracked a health issue. Key qualitative themes emerged that included engagement, acceptability and usability, and structure and content of the program.

**Conclusions:**

Older people use the Internet as a source of health information but have concerns about safe use and quality of information. Users require a credible website that provides personalized information, support, monitoring, and feedback.

## Introduction

Chronic joint pain, often labeled osteoarthritis (OA), is one of the most prevalent health conditions [1,2]. It compromises mobility and physical activity, increases the risk of developing comorbidity [3], reduces quality of life, independence [4,5], and makes people feel anxious and depressed [6]. The socioeconomic cost is high and the consequences associated with chronic joint pain will increase as people live longer, become less physically active, and obesity rates increase [2,7].

Evidence-based guidelines recommend exercise/physical activity, simple analgesics, patient information and advice about self-management, pain coping strategies, and maintaining appropriate body weight [8-10]. Unfortunately, only a small minority of people receive these interventions [11-13], whereas most people are prescribed long-term analgesics despite concerns about safety [14,15], effectiveness, costs [16], and unpopularity [17,18]. Others receive an inappropriately early referral for surgery.

Enabling Self-management and Coping with Arthritic Pain through Exercise (ESCAPE-pain) is a rehabilitation program that integrates information, advice, and self-management and coping strategies with an exercise regimen. The program incorporates behavioral change techniques (BCTs) [19] that challenge people’s erroneous health beliefs about the harmful effects of physical activity on joint pain. It helps them to appreciate that exercise is safe and has wide physical and psychosocial benefits, which results in changes to their behavior by increasing physical activity levels to reduce pain and improve physical function [17,20,21]. ESCAPE-pain is more clinically effective and cost-effective than usual care [22-25] and popular with participants who have pain associated with knee OA [17,18]. Unfortunately, limited clinical resources (such as time, space, and funding) mean that relatively few clinical departments run the program. Therefore, innovative ways are needed to reach the large and rapidly increasing number of people who could benefit from the intervention.

Digital technologies (telemedicine, Web-based programs, mobile phone apps, and so on) are increasingly popular ways of enabling many more people to access information [26-28]. Adapting ESCAPE-pain as a Web-based program would increase access. However, programs are rarely developed with input from end users [29-31] and little consideration is given to the human-technological interaction or context [32]. The resulting products are often unwanted, unusable, “high-tech, low-impact” Web-based programs that users regard as irrelevant and overly complex. These Web-based solutions have poor uptake, lack effectiveness and adherence, and are often unsuccessfully implemented [26-29,33-36]. Adopting a user-centered approach when designing the program might improve the “fit” between human needs and the technology, improving uptake, implementation, and effectiveness [37]. We used quantitative and qualitative research methods, the principles of persuasive technology, and human-centered design to discover what people considered important in the content and design of a Web-based version of the ESCAPE-pain program.

## Methods

### Survey

A postal survey was distributed to 200 people aged 50 years and older with chronic (>6 months' duration) knee, hip and/or back pain who had participated in a trial based on the ESCAPE-pain program in the West of England in the United Kingdom [38]. Of these trial participants, 100 were “experienced” (ie, had undertaken the ESCAPE-pain program, in order to understand what they found useful and necessary to be included in a Web-based program) and 100 people were “naïve” (ie, they had not participated in the program but had received a verbal description of the program’s content and format) in order to understand the needs of a typical person taking part in the Web-based program. Demographic data and Internet use and experience of the participants were collected. The survey posed questions and invited comments about the format and content of the proposed Web-based resource and people’s preferences [39] for features that may improve the effectiveness of Web-based interventions (text messaging, prompts, diaries, support forums, and so on) [37,40-42]. Descriptive analyses were used to describe the survey data.

### Focus Groups and One-to-One Interviews

To gain a better understanding of people’s (dis)likes, opinions, and preferences about the ESCAPE-pain program, how to engage people, and what the Web-based program needed to convey, four focus groups were conducted (n=24 participants in total). Table 1 provides further details of the participant characteristics. Two focus groups involved “experienced” people who had participated in the program (n=11), and two focus groups involved people “naïve” to the program (n=13). Participants were purposively selected from the survey based on characteristics of age, sex, duration and site of pain, and their use of and confidence in using the Internet. Focus groups comprised five to seven participants. The focus groups took place in university or hospital settings, facilitated by one of the authors (JP) using a topic guide (Multimedia Appendix 1), and lasted on average 94 minutes (range 84-105 minutes). Field notes were also taken.

The focus groups and interviews were recorded, transcribed, checked for accuracy, and anonymized, and emerging themes were identified using thematic analysis [43]. Transcripts from all focus groups were coded by JP. The first two focus group transcripts were independently coded by all the authors, who discussed the coding and initial emerging themes. The topic guide was amended to reflect the emerging themes, which was used in the following focus groups. The additional focus group transcripts were independently coded by DC.

**Table 1:** Focus group and interview participant characteristics.

Pseudonym	Sex	Age range (years)	Duration of pain (years)	Site of pain	Experienced or naïve to the ESCAPE-pain program	Confidence in using the Internet	One-to-one interview
John	Male	60-69	>10	Knee and back	Experienced	Very confident	No
Patricia	Female	60-69	>10	Hip and back	Experienced	Neither confident nor unconfident	Yes
Barbara	Female	60-69	1-5	Knee and back	Experienced	Very confident	No
Linda	Female	60-69	1-5	Hip and knee	Experienced	Very confident	No
James	Male	70-79	5-10	Knee	Experienced	Confident	Yes
Mary	Female	60-69	5-10	Multiple	Experienced	Confident	No
Carol	Female	60-69	>10	Multiple	Naïve	Confident	No
Richard	Male	60-69	5-10	Knee	Naïve	Confident	Yes
Betty	Female	70-79	>10	Back	Naïve	Neither confident nor unconfident	Yes
Edith	Female	70-79	>10	Multiple	Naïve	Neither confident nor unconfident	No
William	Male	70-79	5-10	Knee	Naïve	Neither confident nor unconfident	No
Charles	Male	70-79	<1	Knee	Naïve	Unconfident	No
Shirley	Female	70-79	>10	Knee	Experienced	Very confident	No
George	Male	50-59	1-5	Hands	Experienced	Very confident	No
Karen	Female	60-69	1-5	Knee	Experienced	Confident	Yes
Michael	Male	60-69	1-5	Knee	Experienced	Very confident	Yes
Susan	Female	70-79	5-10	Back	Experienced	Very confident	No
Gerald	Male	60-69	>10	Back	Naïve	Confident	No
Gary	Male	60-69	1-5	Knee	Naïve	Confident	Yes
Daniel	Male	60-69	5-10	Hip	Naïve	Confident	No
Donna	Female	60-69	5-10	Back	Naïve	Confident	Yes
Paul	Male	60-69	5-10	Knee	Naïve	Confident	No
Frank	Male	60-69	5-10	Back	Naïve	Very confident	No
Walter	Male	60-69	5-10	Knee and hip	Naïve	Very confident	No

### Prototype Website

**Figure 1:**
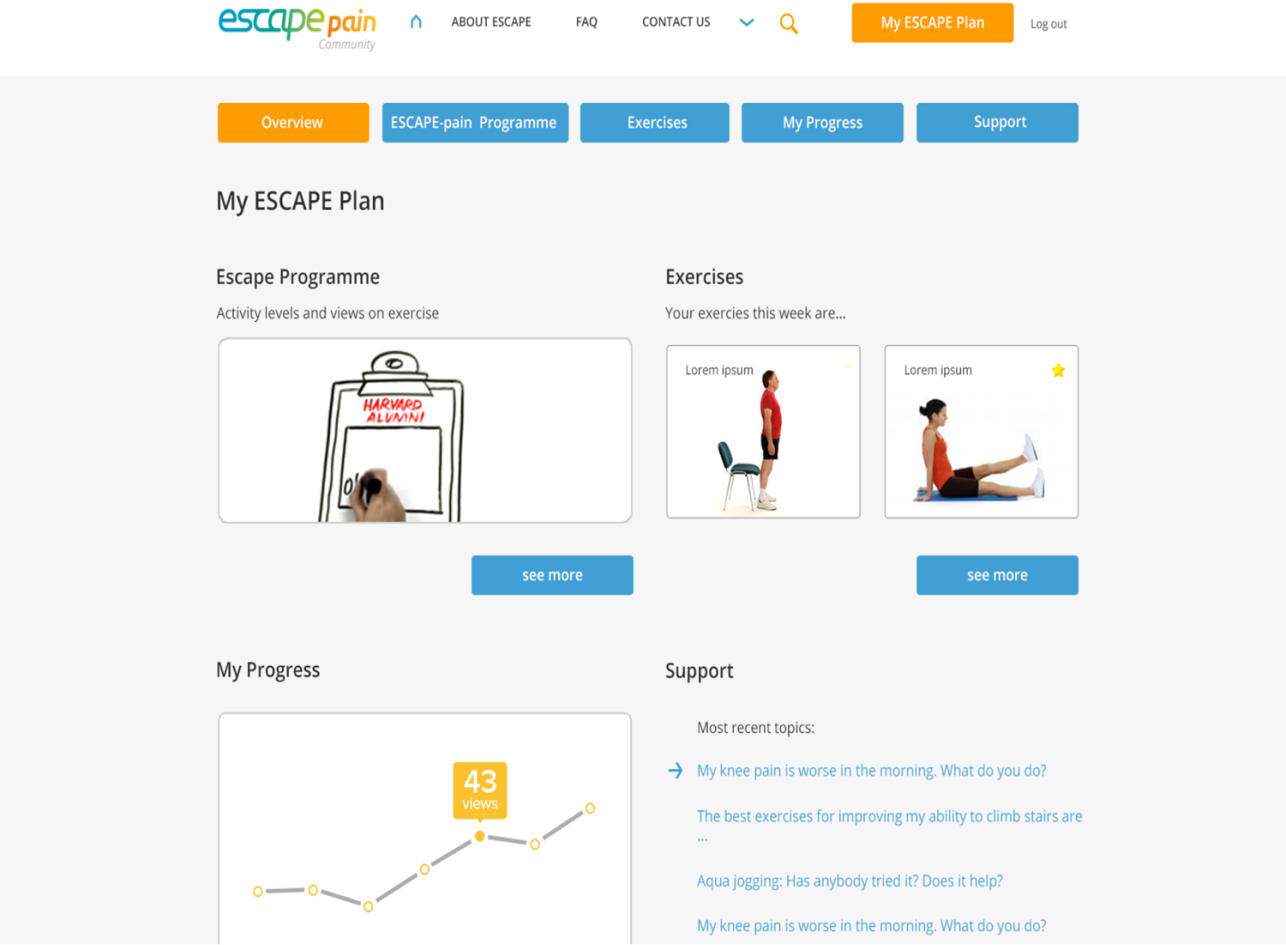
A screenshot of the internal pages of the prototype ESCAPE-pain website.

The results of the survey and emergent themes from the focus groups were used to design a prototype website (see Figure 1). This had limited online function and offline material and resources, consisting of four external pages and four internal pages. The external pages included the following:

- “Home” page gave a brief overview of the ESCAPE-pain program and a Web-based promotional video explained the concept of the program.
- “About ESCAPE” explained the history of the program and its potential benefits, provided information about the website's contents, videos of participant endorsements and testimonials, and information of partner organizations.
- “Frequently Asked Questions.”
- “Contact Us” had a registration section and ways to contact the ESCAPE-pain team.

The internal pages consisted of the following:

- “My ESCAPE Plan” page provided an overview of the 12 modules that compose the program, such as physical activity, exercise, goal setting, action plans, pacing, drug management, diet, home exercises, understanding pain, pain management, and relaxation.
- “Exercise” consisted of a library of “easy,” “medium,” or “advanced” exercise videos suitable for people with joint pain, which they could watch and which provided instructions on how to perform the selected exercise.
- “My Progress” provided an example of how people could input data and view graphical feedback on the amount of exercises they performed, as well as their activity levels, mood, and so on.
- “Support” provided an example of a forum where participants could join to discuss joint pain–related issues, with links to other Web-based resources.

### “Think Aloud” Semistructured Interviews

The prototype website was then tested by eight participants using semistructured interviews and a modified “think-aloud” technique [44]. The participants were purposively selected based on the key characteristics of age, sex, duration, site of pain, and their confidence in using the Internet. JP conducted the interviews at participants' homes, which lasted on average 107 minutes (ranging between 78 minutes and 142 minutes). Participants were shown the prototype Web-based version of ESCAPE-pain and asked questions using a topic guide developed from themes emerging from the survey and focus groups and features of the prototype website (Multimedia Appendix 2). Table 1 indicates those participants who were involved in the “think aloud” interviews.

The interviews were analyzed using a thematic analysis [43]. JP coded all the interviews, the first two interview transcripts were independently coded a second time by MH, DC, and NW, then each member coded two additional transcripts so that each interview was coded independently by three authors. The final themes were then mapped to the behavioral change taxonomy [19]. The findings from the focus groups and interviews are presented together.

**Ethics**

Ethics approval was obtained from the National Research Ethics Service Committee South West-Central Bristol (Research Ethics Committee reference 11/SW/0053). All participants received information about the project and gave signed informed consent.

## Results

### Survey

The survey response rate was 42% (83/200). The demographics of the respondents reflect the demographics of people with OA, predominantly female (56/83, 67%), mean age was 67 years (range 53-84 years), over two thirds were retired and had pain in several joints for a prolonged time (Table 2).

Most respondents (71/83, 86%) had used the Internet, of whom 83% (59/71) used it daily and almost exclusively at home (99%). Most people felt confident or very confident using the Internet (49/71, 69%) with personal computers, tablets, laptops and/or mobile phones. Although more than three-fourths of the people used the Internet to search for information about health problems, few (14/71, 20%) had used this information to manage their condition (Table 3).

**Table 2:** Participant demographics.

Participant demographics		Survey (n=83), n (%)	Focus group (n=24)	Interviews (n=8)
**Sex**				
	Male	27 (33)	12	4
	Female	56 (67)	12	4
**Age, years**				
	50-59	10 (12)	1	-
	60-69	50 (60)	16	6
	≥70	23 (28)	7	2
**Site of joint pain**				
	Knee	17 (21)	5	3
	Hip	1 (1)	1	-
	Back	13 (16)	3	2
	Other	6 (7)	1	-
	Multiple	46 (55)	14	3
**Duration of joint pain, years**				
	<1	5 (6)	1	-
	1-5	27 (33)	6	3
	5-10	26 (31)	9	3
	>10	23 (28)	8	2
	Unknown	2 (2)	-	-
**Trial group**				
	Experienced	33 (40)	11	4
	Naïve	43 (50)	13	4
	Unknown	8 (10)	-	-

**Table 3:** Internet use and online activity.

Internet use and online activity		Survey (n=71), n (%)	Focus group (n=24)	Interviews (n=8)
**All devices used (data represent use of multiple devices)**				
	PC^a^	42 (59)	18	7
	Laptop	30 (42)	16	7
	Tablet	33 (46)	13	3
	Mobile phone	21 (30)	9	5
**Confidence**				
	Very confident/confident	49 (69)	18	6
	Not confident/unconfident	16 (22)	5	2
	Unconfident	4 (6)	1	-
	Unknown	2 (3)	-	-
**Frequency of use**				
	Daily	59 (83)	19	7
	Weekly	7 (10)	4	1
	Less often	5 (7)	1	-
**Searched Internet for health information**				
	Yes	55 (77)	18	6
	No	16 (23)	6	2
**Watched/read someone's health-related experience**				
	Yes	24 (34)	8	3
	No	47 (66)	16	5
**Contributed to online discussion**				
	Yes	3 (4)	2	1
	No	68 (96)	22	7
**Tracked health-related symptoms**				
	Yes	16 (23)	6	3
	No	55 (77)	18	5
**Used an online resource to manage an existing health condition**				
	Yes	14 (20)	9	3
	No	57 (80)	15	5

^a^PC: personal computer.

### Focus Groups and Interviews

From the focus groups and “think aloud” interviews, three key themes emerged that influenced people's use of the proposed website (Table 4): (1) initial and sustained engagement with the website, (2) acceptability of a Web-based exercise-based self-management program, and (3) content/structure of the program.

**Table 4:** Features to encourage engagement with and improve effectiveness of a Web-based program mapped onto the behavioral change taxonomy [19].

Theme	Subtheme	Supporting quote	Taxonomy
Engagement	Credibility	“...you go to sources that you know are genuine...For medical information I would always go to the NHS website...” Mary “...it's got to be recommended to you by a professional...” Paul “...I am very wary about the web and medical problems...but this is one that was recommended by the GP...” Daniel “...[research information] adds weight to the thing...communicates integrity I think it's a serious piece of work...” Donna “...not sponsored by drug companies...” Gerald	9 Comparisons of outcomes 9.1 Credible source
	Patient testimonials	“...I think [patient testimonials] explained the purpose very well...it showed a good cross section of different people doing different exercises...I think I would be encouraged to pursue it further...Makes you realize that people can get good improvement. Yes that these are people who have been through the program and have found benefit and its much improved their quality of life...it makes you realize you are not alone...” Patricia “...I always skip over those sort of things...” John “...I don't actually I like reading other people's experiences because it somehow makes it feel more real you know...this is really flippant but if it's a recipe I have looked up on line and you know a hundred people have said oh yeah that was absolutely brilliant I will give it five stars or something then you think well great I'll do that...” Mary	6 Comparison of behavior 6.1 Demonstration of the behavior 6.2 Social comparison 6.3 Information about others' approval
	Social identification	“...this program is designed for later life people isn't it? So it should have them in it I think. Not young people who can do it easily and no pain, but people who are actually finding it hard...” James “...I watched a little clip a couple of years ago because I had labyrinthitis and that was really awful and seeing this girl saying how she had experienced it and how she came through it and she was obviously ten time worse than mine...” Barbara	
Acceptability and usability	Aesthetics	“...something nice and bright that would attract you to the site, you know to attract you...very self-explanatory...” Betty “...plying a very positive image...” Gerald “...its plain and simple its not too fussy it tells you what you want to know...” Patricia “...well I think simplicity is the best thing...it's got to be interesting...” George “...big fonts, nice and clear, plain simple language, hasn't got thousands of links on it, asks intelligent questions and leads the user to the information you get to what you want within about 2 or 3 screens...” John “...[not] loads and loads of advertisement...” Richard	
	Functionality	“...something simple you know so people can just click in and find out what they want...” Betty “...easy to navigate through...navigation is the key...” Mary “...I am not going to spend hours trawling through stuff but if there's a star or something like that that says this that and it you know captures the attention because there is a need then you can go into that and it opens up the bit that you need...” Karen “...you don't want hundreds of links...” Richard “...one of the problems with links as well is that you link to something and that gives you a link to something else and before you know where you are you can't remember how you got there...” Carol	
	Registration	“...oh not something you have to register for...” Barbara “...I hate that you have to put passwords in...and all that malarkey just get the flipping information...” Mary “...certainly not open an account because that always sounds like money to me...” James “...people ought to log on they should sign up I mean because I think it would help enormously the feeling of actually belonging to something...” Michael	
	Technical capability	“...my mother-in-law is 90 plus she has never switched a computer on she doesn't know how to use it...she will never use that website...” Gerald “...this problem of aged people and computers is going to drift away...maybe it's only a problem possibly for another 15 years...” Daniel	
Content and structure of program	Information and advice	“...[information] needs to be in a visual form rather than a written form...an executive summary of the sequence rather than the detail of the sequence so you would read the executive summary and then get into the detail...” James “...if I got them at my bedside cabinet or pinned up on the fridge, I will always remember to do them...” Shirley “...for someone that's coming in and using it for the first time it would be nice to if its presented sequentially if someone's going back to it they want to go directly to there...” John	4 Shaping knowledge 4.1 Instruction on how to perform a behavior 7 Associations 7.1 Cues/prompts
	Exercise	“...a nice little looped video wouldn't they, demonstrating the exercise...” John “...it's clear what the exercise is, and how it's going to benefit you, and what you might do wrong, and how that is going to affect you...” Linda “...how often should these exercises be done, and how many repetitions all the things that we had in the class that we went to...” Mary	4 Shaping knowledge 4.1 Instruction on how to perform a behavior 5 Natural consequences 5.1 Information about health consequences
	Personalized	“...need for the website to be an individual's website...they can have you know their own diary, they can have their own records...” Michael “...it has to be personalized to you otherwise you know why do you bother to turn it on...it shows you what you have done so that you can when you have done something you can actually tick it and you get something to show you have done something...this is the goal setting... right so you have got your goal setting you are achieving so and so there's your action plan have you done it and is there something along the line that shows you, you have done something...” Gary “...if I was going to start my activity and exercise with it I would want the goal setting...” Donna ”...set your target and get a little bit better each time you do it, or each week you do it, sort of build yourself up...” Betty	1 Goal setting and planning 2 Feedback and monitoring 3 Social support
	Monitoring	“...if there was sort of a little personal diary where you could say, ‘Tuesday did 10 of this and 15 of that,’ then maybe some monitoring person says, ‘well next week you should do 20 of those and 25...I would definitely want to monitor my progress...” Patricia “...you have got your exercises, you have got my progress. I like that because you can then monitor where you are and you have also got support...I can look at that and feel I have achieved...I like to see the chart because the progress bit like I did for my weight so I would like to see that you know because it would then tell me you know have you done them this week or haven't you done them...you can look to see well three months ago I could only do so and so ooh, look now I can do this so again its goal setting if you like, but I mean its yeah its moving forward all the time...” Gary “...I don't know whether I would monitor myself...” Betty	2 Feedback and monitoring 2.2 Feedback on behavior 2.3 Self-monitoring of behavior 2.4 Self-monitoring of outcome behavior
	Peer support	“...a members site you can have a blog....so you could actually contact then talk to people with similar problems...” Gerald “...it could be interesting to see how they are getting on and support each other you know if they have had a down day or something has gone wrong sort of be able to be a bit supportive...I would like to be in contact with other people...to see how other people do the exercises it gives you a good idea of whether you are doing it right...[has she used forums?] very rarely...it was too time consuming...I wasn't sure that it was being any help for me...” Patricia “...you get somebody else's feedback on what they do to get rid of their knee pain in the morning. I mean it might not suit you, you could try it and if it doesn't suit you perhaps you could look for something else that would help you, do you know what I mean?...” Betty	3 Social support 3.1 Unspecified 3.2 Practical 3.3 Emotional
	Professional support	“...you need some personal physio input to start with...you need to be doing that first set of exercises with a physio so you get direct feedback...eight weeks, or whatever it is, you can then go off and do it on the internet...not standing on its own as a substitute [for a face-to-face program]...” James “...I think I would want something personal...something more than just being told to go and look at a website...you want reassurances I guess so if you have been doing it for two months then nothing has happened then you actually want to know how long you should be doing the exercises...” Barbara “...I think it would be difficult to give people the confidence to accept [an Web-based program] as a sole treatment avenue...” John “...I would feel less confident about the information than if I had had some physical contact with doctor, physio something else beforehand and I think probably most people would think along those lines, you know, like your concerns about a replacement [for a face-to-face program] it doesn't feel that comfortable...” Linda “...websites that you can go on that there is literally somebody at the other end so you can type in a question and then you get an answer back...” Paul “...an email to say have you done your exercises this week...” Patricia “...I would need somebody who actually knew about pain and knew about exercise...” Michael	2 Feedback and monitoring 2.7 Feedback on outcomes of behavior 3 Social support 3.1 Unspecified 3.2 Practical 3.3 Emotional

### Engagement

Finding a health-related website usually results from a purposeful search for information about a specific health issue, for example, joint pain. However, people are often skeptical and mistrustful of information they find online. In our study, people wanted to be directed to Web-based resources by a trusted “source” such as a health care professional [45] or credible organization (ie, a statutory health body) [46-48]. They were mistrustful of commercial websites with advertisements, which they saw as promoting goods for financial gain with little regard to whether it will help the patient [46,47]. The website needed to capture people's attention visually, with content that was obviously relevant and created a positive association between the website and the health benefits it could bring [46,47]. Important determinants for engagement with the website were video testimonials of people discussing their experiences of participating in the program, with personal and social traits that potential users could identify with [46,47].

### Acceptability and Usability

In general, people thought a Web-based ESCAPE-pain program could be an effective way to help them self-manage their condition. However, they thought there were limitations to how well they could do this and wanted to know when to seek advice from a health care professional, especially in regard to the exercise regimen. Some people were willing to accept the Web-based program as a replacement for face-to-face contact, while others saw it as an adjunct to the supervised face-to-face program that would help them sustain benefit and motivation after completing the program.

Acceptability partly depended on people's technical ability in using Web-based resources. Regular Internet users, familiar with technology and using Web-based resources to manage a health condition, were most accepting of the proposed website. Infrequent users, unconfident in their ability to use the Web-based resource correctly, thought they were less likely to use the website.

The website had to be aesthetically pleasing, which was defined as simple, bright, attractive, and interesting. However, people's aesthetic preferences (ie, color schemes, layout, icons, dropdown menus, scroll bars, and so on) varied greatly and were frequently mutually exclusive.

Navigation around the website had to be easy and intuitive so people could find what they wanted quickly, and information had to be easy to understand and free from jargon and acronyms. Internal and external links needed to be working, obvious, self-explanatory, and up to date. The website also needed to be optimized to work on different devices (ie, laptop, mobile phone, tablet), platforms (ie, Android, iOS), and Web browsers.

### Content and Structure of a Web-Based ESCAPE-pain Program

People wanted information and advice tailored to their personal needs, which was easy to understand, was practicable, and brought tangible benefits [48]. To surmount the difficulties of individuals having different needs and preferences, they suggested each person could have “their own website” that stored personalized information and advice, allowed for creation and adjustment of personal goals, as well as monitoring and recording of progress and achievements. When it was suggested that people might need to register to use the website to its full potential (ie, monitor, record, and chart progress and enable provision of targeted personalized support) many people were uncomfortable [48]. Even if registration was free they thought it might be a barrier to some people using the website because they would be suspicious about the reasons for registration. People also expressed concerns about the difficulty of remembering usernames and passwords.

In general, people who had “experienced” ESCAPE-pain thought a Web-based version should replicate the program. People “naïve” to ESCAPE-pain asked for broader information to explain their joint pain and how to cope with its effects. For example, they wanted to know about the following: why they get pain at night; home adaptations or assistive devices that might help them cope; what the “placebo effect” was; and the association between alcohol and joint pain. People also wanted information about the effectiveness of diet and complimentary medicines (ie, fish oils, food supplements, acupuncture) in preventing or relieving joint pain. They thought this might be best presented as a brief summary of essential information, educational videos, supplemented with printable leaflets for reference and linked to reputable external sources for more in-depth information [39].

When delivered face-to-face in a clinical setting, the educational sessions of ESCAPE-pain are presented in a specific sequence. However, people thought that online this would be very restrictive and cumbersome; they wanted easy access to relevant information when it was most appropriate rather than having to navigate through “irrelevant” information. For example, people wanted to find information about dealing with an exacerbation of pain when they were in pain without going through sessions discussing weight control and diet.

#### Exercise

The ESCAPE-pain program focuses on the benefits that physical activity and exercise have on pain, mobility, and physical and psychosocial function. To achieve this, people undertake a progressive exercise regimen, starting off with simple exercises and gradually increasing both the number of repetitions and the quality of performance, while adding more challenging exercises as they improve. When delivered face-to-face, the exercises are supervised by an appropriately qualified professional. The Web-based program would necessitate participants to choose, perform, and progress their own exercise regimen. To achieve this, people thought they would need detailed information about exercises and exercising—how to choose appropriate exercises, how to perform them correctly, how many repetitions they should do, how often, what they should feel, and how they would know whether they were doing an exercise incorrectly or potentially causing harm. Videos showing people exercising with instructions on how to do them and how often were well received. Many people said they would be happy to try the exercise regimen, but some were concerned about performing the exercises without supervision and wanted to know when they should seek advice from a health care professional.

#### Monitoring

Many people wanted to document their progress to evaluate the usefulness of the program. Monitoring was also seen as a good way to engage people with the Web-based program. However, views on what should be monitored, how, and when varied. Most people thought online diaries documenting levels of pain and physical activity would be essential. Mood and medication were also suggested but considered less important. People who were more engaged with self-managing their condition wanted to monitor symptoms frequently, whereas others thought they would only likely monitor themselves occasionally, when reminded, or if it was compulsory. In some cases, monitoring was considered an intrusive burden requiring time, effort, technical ability, and appropriate hardware.

#### Support

Another way of maintaining engagement and interaction with a website is by providing support. People were particularly keen to have some form of support from a health care professional (ie, physiotherapist, general practitioner) who could monitor progress, guide progression, reinforce health messages, and provide reassurance, motivation, and encouragement. Peer support through online communities (ie, forums, blogs) where people could share experiences to learn from and support other people with joint pain was seen as a positive feature, although users thought it would need to be moderated to prevent inaccurate and inappropriate postings.

## Discussion

Using persuasive technology and human-centered design we investigated what features help people discover, engage with, and benefit from a Web-based program to manage chronic joint pain. If endorsed by credible organizations, some participants would be happy to accept a Web-based program as an alternative to a face-to-face program, although others did not believe it could adequately replace a face-to-face program aiming to change entrenched beliefs and behaviors. To be effective, information needs to be easy to understand and personalized for each user. People want to monitor their progress, receive feedback and guidance from a health care professional, and learn from others with similar problems. Feedback on what this might entail and the way it would be delivered varied considerably, and could be mutually exclusive, but overall it needed to be simple to minimize burden and technical requirements and, above all, protect people's privacy.

Increasingly, older people use the Internet as a source of health information [49] and accept it as part of their management [39], especially if it supplements—rather than replaces—personal care [50]. However, few utilize online information to fully self-manage their condition, often doubting the accuracy of the online information and the motives of commercially sponsored websites [46]. Approval and endorsement by health care professionals, coupled with testimonials by people that users could identify with, are very influential in encouraging people to visit a website and convincing them to engage with it and implement online advice [46,47,50].

Pain is a ubiquitous, multifaceted problem that evokes unique and varied personal experiences and consequences. It often induces health beliefs that result in harmful behavior. For example, people with chronic joint pain commonly experience pain during physical activity. They erroneously surmise activity-related pain is harmful and reduce or avoid activities to prevent causing pain and harm—“fear-avoidance behavior.” Unfortunately, fear avoidance results in muscle weakness that can exacerbate pain, functional limitation, and disability [51,52]. Face-to-face behavioral change programs, such as ESCAPE-pain, incorporate BCTs to challenge these erroneous health beliefs and promote healthier behavior, namely, the role of physical activity in the management of chronic joint pain. Effective Web-based behavioral change programs need to incorporate the BCTs that make face-to-face programs effective [19,53-57].

Altering behavior involves several steps. First, people need to be convinced of the consequences of poor health behavior (inactivity) and good behavior (activity). They then need clear, unequivocal information and instructions about what (not) to do, demonstrations of how to perform specific exercises, how often, and what to expect. Knowing why and how to exercise helps form strong “implementation intentions” [58]. Unfortunately, good intentions are often not turned into action. Bridging this “intention-behavior gap” requires people to form “action plans” (which specify behaviors they want to perform, where, when, and how) and “coping plans,” which raise awareness of their personal strengths, weaknesses, and situations that could undermine behavior and prepare a coping strategy to overcome these barriers [59-61]. Behavioral change is also facilitated if people self-monitor their behavior and compare it with what is expected of them (their level of physical activity compared with how physically active should they be) and this includes factors such as goal setting, feedback, and support of performance and progress [19,53,57]. Although information is usually provided in Web-based behavioral change programs, the incorporation of methods to help people construct intentions to change, such as goal setting, action plans, and interactive feedback and support, are more difficult and consequently do not often feature in many Web-based programs [56].

Our study highlights the importance of BCTs. Participants intuitively appreciated that they require in-depth, accessible information to explain and demonstrate why and how to exercise correctly. Additionally, users value self-monitoring and self-regulation, feedback, support, and encouragement, all of which can be personalized to each person's unique circumstances to make it effective and sustain engagement [55]. However, different people have varying preferences, which are often mutually exclusive. Achieving personalization is extremely difficult as it increases user time and effort burden, technical requirements, cost, and the need to register. These are features people identify as barriers to engaging with Web-based resources. Moreover, patient forums that are often advocated to facilitate health care professional and peer support [55] were rarely used by our survey respondents and the respondents in other studies [62,63].

### Strengths of the Study

The survey response rate (83/200, 42%) was reasonable and allowed us to recruit focus groups and interviewees representative of people with chronic joint pain. Using participants who were “experienced” to an ESCAPE-pain based program enabled us to understand what they considered important to convey and how to convey it, while using people “naïve” to the program enabled us to understand what more “typical” people with no prior experience of the program would need in order to apply it.

### Limitations of the Study

Although participants were purposely sampled to reflect the wide opinions of people, only one person in the focus groups and none of the participants selected to test the website were unconfident about using the Internet. This means that Internet users lacking confidence were underrepresented. However, this did not have a significant impact on our findings because the prototype tested had limited functionality and was problematic even for experienced, confident Web users. Another limitation of the study was that the prototype website could only be displayed on a laptop or desktop computer, meaning it was difficult for users who are familiar with other devices, such as tablets, to feel as confident when testing the website and giving their opinions.

### Conclusions

As more people live longer, and obesity and sedentary lifestyles increase, the prevalence of chronic joint pain will increase [1,2]. Web-based programs have the potential to reach the large number of people requiring help, but historically the uptake of, effectiveness of, and adherence to Web-based interventions are notoriously poor [64-66]. Persuasive technology and human-centered design and business modeling can inform the design and content of engaging, effective Web-based behavioral change programs.

## Appendix

Multimedia Appendix 1 Focus group topic guide.

Multimedia Appendix 2 Interview topic guide.

## Abbreviations

BCT: behavioral change technique
ESCAPE-pain: Enabling Self-management and Coping with Arthritic Pain through Exercise
OA: osteoarthritis
