# Supplementary material for: Developing a Web-Based Version of An Exercise-Based Rehabilitation Program for People With Chronic Knee and Hip Pain: A Mixed Methods Study
Source: JMIR Res Protoc. 2016 May 19;5(2):e67. doi: 10.2196/resprot.5446 (PMC4891573; doi:10.2196/resprot.5446)
Supplement: Multimedia Appendix 1 [file resprot_v5i2e67_app1.pdf]

Appendix A
Focus group to inform the early development for an online activity and self-management programme for osteoarthritis – OA intervention topic guide

Individual consent
Background information
Aim and objectives
Ground rules
Final consent

Introductions
- How long have you experienced joint pain and where does it affect you?

Intervention
- What did you learn from the OA programme?
- How have you used the information provided from the OA intervention? Is so how?

Internet
- What are your favourite websites and why do you like them?
- Have you looked online for information about osteoarthritis?
- Have you used any other online resource to manage health conditions?
  Tracking symptoms, weight loss, stopping smoking, increase exercise/activity, diabetes resource

Future website development
- What aspects of the programme would work well or not so well on the internet?
  - *Exercises, information, advice, diaries*

- What information would you like to see on a future website for an osteoarthritis intervention?

  - *Joint pain and benefit of exercise, goal setting and action plans, pacing activities, healthy diet, ice and heat, anxiety, mood and pain, relaxation techniques, managing flare ups, exercising in the long term*

- How would you like this information presented?

  - *Textual, visual, audio*

- What would information would you like on the homepage?

- What information would you like about exercise/activity on the website?

  - *Explanations of the exercises, length of time exercises take, videos, length of videos, exercise demonstrators*

- What do you think about having access to peer support on the website?

  - *Forums, comments boxes, rating systems of advice*

- What other features would you like on the website?

  - *Text alerts, e-mails, other people's OA experiences, membership, log in, links, printable information*

- How can we best personalise information about osteoarthritis for you?

- What information would you like us to monitor if any?
- How can we best monitor your progress on the website?
    - *Diaries*
- Would you want access to professional support from the website? If yes, how …
- What would you think if your GP directed you to a website to find out more about your problem and self-manage it? Would you follow their advice?

Closing:
Thanking re information given, reflection on what was said, and other questions?

Consent:
Reiterate confidentiality and thank
