# Supplementary material for: Developing a Web-Based Version of An Exercise-Based Rehabilitation Program for People With Chronic Knee and Hip Pain: A Mixed Methods Study
Source: JMIR Res Protoc. 2016 May 19;5(2):e67. doi: 10.2196/resprot.5446 (PMC4891573; doi:10.2196/resprot.5446)
Supplement: Multimedia Appendix 2 [file resprot_v5i2e67_app2.pdf]

Appendix B

Topic guide: Interview

Background, Confidentiality and Consent

External pages

Home page
What do you think about the homepage? What do you like or dislike about it? What other information would you like to see on the homepage? What key information would you like to see on the homepage?
Structure, clarity, images, use of videos, branding, partners

Promotional Video
What do you think about the promotional video? What do you like or dislike about it? What information would you like on a promotional video for the website?
Clarity, additional information

Testimonials
What do you think about the testimonial information?
Appropriate, relevance, people

Registration
What do you think about registration to the website? What other questions would you like to see or be removed from the registration section? What are the advantages/disadvantages of registering with the website?

About ESCAPE
What do you think about the information? Do you feel you understand the purpose of the internet programme? Do you have any concerns about the programme? Is it clear who supports and endorses the programme? Is it necessary to have the supporting information on the website?
Clear, appropriate

FAQ
What do you think about the questions? Are there any other questions you would like to see in this section?
Relevance

Contact us
Would you contact the website? Who would you want to contact? How soon would you expect a response?

Internal pages

My Escape plan
What do you think about the information in the ESCAPE plan? Is there any other information you would like to be displayed? *Clear, visual display of information, functionality*

ESCAPE-Pain programme
What do you think about the Escape-pain programme? What do you think of the programme? Do you like how the programme has been presented to you? How would you go about completing the Escape pain programme? Would you like complete the programme in a set way or would you like to choose

the modules you want to complete? How would you like the self-management module information be presented to you?
Visual line to display modules, tick function, videos, other information,

Exercises
What do you think about the exercises? Are the instructions clear? Would you find a search function helpful? Would you like to document your progress, and if so how?

Reps and sets, exercise abilities, language (easy, medium and hard)

Progress
What do you think about the progress section of the website? Is there any other information would you like to see here? How often would you use this functionality? What do you think about the way the information to track your symptoms is presented? Would you like to set specific goals? What would these be?
Type of information displayed, monitoring information

Support
What do you think about the support section of the website? What other content would you like to see in this section?
Forums, chat room, blogs, moderation

Forums
Could you tell me about your previous use of forums, if any? How would you react to medical advice given on a forum? How much confidence do you have around advice from other participants? What would you do if you saw inappropriate comments/advice? Who would you like to communicate with?

Usability
How easy or difficult is the website to use? How clear are the website instructions?

Aesthetics
What do you think about the way the website looks?
Layout, ESCAPE-pain branding, visual information, pictures

Future improvements
How can we improve the website? What other terminology would you prefer to see? What other information would you like to have? What other functions would you like on the website?

Confidentiality, Consent, Thanks and Close
